# Cardiac magnetic resonance imaging for noninvasive assessment of cardiac allograft during the follow-up of patients after heart transplantation

**DOI:** 10.1186/1532-429X-15-S1-P265

**Published:** 2013-01-30

**Authors:** Maria Fernanda Braggion Santos, Jan Simpfendörfer, Hugo A Katus, Henning Steen

**Affiliations:** 1 Universitatsklinikum Heidelberg, Heidelberg, Germany; 2 School of Medicine of Ribeirao Preto University of Sao Paulo, Sao Paulo, Brazil

## Background

Heart transplantation (HTX) is an established life-saving treatment for patients with end-stage heart failure. However, several injuries can compromise cardiac allograft function over the years after HTX, i.e. organ rejection, transplant vasculopathy or infections which can lead to myocardial fibrosis, cardiac remodeling and ventricular dysfunction. Non-invasive late gadolinium contrast enhanced MRI (LGE-CMR) is able to identify myocardial tissue alterations as well as morphological and functional changes. To date, no longitudinal follow-up CMR data on myocardial function and tissue characterization exists on patients after HTX. Therefore, we sought to evaluate longitudinally functional and morphological changes after HTX employing non-invasive LGE-CMR.

## Methods

61 HTX patients were included in the study and were scanned twice after HTX (scan 1, scan 2, Table 1). 48 patients had coronary angiography within 4 weeks after CMR. Cine CMR with 32 channel image acquisition and vector-ECG gated short axis, two and four chamber cine slices with parallel image acquisition covering the entire left ventricle (LV) were acquired using a regular SSFP sequence on a 1.5T Whole Body MRI scanner (Achieva 1.5T, Philips Medical Systems). LGE-CMR (Gadolinium-DTPA:0.2 mmol/kg, Magnevist) was performed and analyzed by two experienced blinded observers. The presence and patterns of LGE (infarct-typical and -atypical) as well as left ventricular morphology and function were assessed. Cardiac allograft vasculopathy (CAV) stenoses were divided into four stages:0=no;1=mild;2=moderate;3=severe stenosis. The Student t-test for paired samples and the McNemar's test were used to compare the group in different periods. P-values ≤ 0.05 were considered statistically significant.

**Figure 1:**
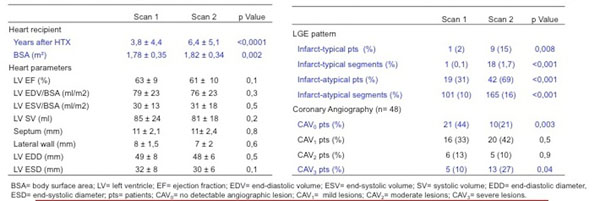
Comparison of CMR Morphology and Function, Presence and Pattern of LGE and Presence of CAV in 2 Different Time Perios after HTX

## Results

The mean time after HTX was 3,8 years for scan1 and 6,4 years scan 2. Although there was a significant increase of LGE for both patterns considering the number of patients affected and numbers of segments involved based on the AHA-17-segment-model, there was no statistical difference in the left ventricular morphology and function. Coronary angiography revealed a statistically significant difference in diagnosed cardiac allograft vasculopathy (CAV) only for CAV0 and CAV3 but not for intermediate lesions (CAV1/2, Figure 1).

## Conclusions

The increase of both infarct-typical and -atypical LGE patterns after HTX can be considered as a result of several injuries over the years after HTX. The lack of changes in cardiac morphology and function may be explained by the strict surveillance and early treatment of organ rejection, infections and the advances in the immunosuppressive therapy.

## Funding

none

